# Early vascular ageing biomarkers in osteoporotic outpatients: a pilot study

**DOI:** 10.1038/s41598-020-76427-1

**Published:** 2020-11-10

**Authors:** Agostino Gaudio, Anastasia Xourafa, Luca Zanoli, Rosario Rapisarda, Antonino Catalano, Salvatore Santo Signorelli, Pietro Castellino

**Affiliations:** 1 Department of Clinical and Experimental Medicine, University of Catania, Catania, Italy; 2 Department of Clinical and Experimental Medicine, University of Messina, Messina, Italy; 3 UO di Medicina Interna, Policlinico “G. Rodolico”, Via S. Sofia 78, 95123 Catania, Italy

**Keywords:** Endocrine system and metabolic diseases, Cardiovascular biology, Predictive markers

## Abstract

Osteoporosis and atherosclerosis are significant public health problems that often coexist, especially in the elderly. Although some studies have reported an age-dependent relationship, others have suggested a causal relationship between osteoporosis and atherosclerosis. The aim of our study was to evaluate the cardiovascular risk in a population of patients with osteoporosis by measuring carotid intima-media thickness (cIMT) and carotid-femoral pulse wave velocity (cf-PWV). A total of 58 patients with osteoporosis and an equal number of healthy control subjects were enrolled. All subjects underwent (1) a bone densitometry examination using dual X-ray absorptiometry, (2) a vascular evaluation for the measurements of cIMT and cf-PWV and (3) a blood sample for the evaluation of lipids and phosphocalcic metabolism. Patients with osteoporosis had a significant increase in cIMT and cf-PWV. There was also a significant inverse correlation between the femoral neck BMD and cf-PWV values. In conclusion, osteoporotic outpatients have earlier vascular ageing, with an increase of arterial stiffness. These data support a possible association between osteoporosis and atherosclerosis independent of age.

## Introduction

Osteoporosis is a systemic bone disease characterised by impaired bone strength that predisposes to a high risk of fracture^1^. It represents a disease of social relevance, and its incidence increases with age^2^. Numerous studies have reported that patients with osteoporosis have an increased risk of developing atherosclerosis and cardiovascular disease^3^. Although some studies have reported an age-dependent relationship^4,5^, others have suggested a causal relationship between osteoporosis and cardiovascular disease^6,7^. In a recent meta-analysis of 25 studies with 10,300 patients conducted by Ye et al.^8^, the incidence of any atherosclerotic vascular disease was significantly increased in subjects with low bone mineral density (BMD) compared to those with normal BMD, with an odds ratio (OR) of 2.96 and after adjustment for the various cardiovascular risk factors. These data were confirmed by a subsequent meta-analysis (with 11 studies) conducted by Veronese et al.^9^ The authors reported that subjects with low BMD had an increased risk of developing cardiovascular disease during the follow-up period with a hazard ratio (HR) of 1.33. There is also evidence that patients suffering from cardiovascular disease have an increased risk for the development of osteoporosis and fragility fractures^3^. In a study conducted in 32,000 Swedish twins, the authors observed that the subjects, after the diagnosis of heart failure, stroke, peripheral arterial disease or ischemic heart disease, encountered a hip fracture, with HRs of 4.4, 5.1, 3.2 and 2.3, respectively^10^.

There are multiple possible pathophysiological links between osteoporosis and atherosclerosis or cardiovascular disease regardless of age. Apart from the fact that the two diseases share common risk factors, such as high blood pressure (BP), diabetes mellitus, kidney failure, smoking, alcohol abuse and low levels of physical activity, osteoporosis and atherosclerosis also share common potential pathogenic mechanisms, such as oestrogen deprivation; abnormal homocysteine levels; inflammatory processes and cytokine networks; and altered levels of total and hypocarboxylated osteocalcin, vitamin D, vitamin K, lipid oxidation products, fetuin-A and fibroblast growth factor 23^11^. In addition, the vascular calcification process seems to share some common markers with bone mineralisation, such as bone alkaline phosphatase, bone matrix proteins, the osteoprotegerin (OPG)/receptor activator of NF-κB (RANK)/RANK-ligand (RANKL) system and WNT/ß-catenin signalling and some transcriptional factors that regulate osteoblast differentiation^11^. To date, several studies have been published about the possible role of sclerostin, a soluble inhibitor of WNT signalling, and OPG as early markers of atherosclerosis^12,13^. Despite much research, the mechanisms of this increased atherosclerotic process in patients with osteoporosis have not yet been completely clarified.

The aim of this study was to search whether there is a relationship, independent of age, between osteoporosis and two early markers of atherosclerosis: common carotid artery intima-media thickness (cIMT) and carotid-femoral pulse wave velocity (cf-PWV).

## Methods

### Study subjects

This cross-sectional study was conducted at the Department of Clinical and Experimental Medicine of the University of Catania in Italy. The study included patients with osteoporosis according to the World Health Organization (WHO) classification (T-score ≤  − 2.5 standard deviations (SDs) at the lumbar or femoral level)^14^ or with osteoporotic fractures independent of their BMD and a group of healthy subjects not affected by osteoporosis. Subjects with malignancies; chronic renal insufficiency (serum creatinine > 1.4 mg/dl) or liver insufficiency; malabsorption syndromes; cardio or cerebrovascular diseases; diabetes mellitus; or who were already being treated for osteoporosis or with drugs interfering with bone metabolism (e.g. glucocorticoids), with the exception of calcium and vitamin D, or on lipid therapy (statins or fibrates) or on therapy with antiplatelet agents or anticoagulants, were excluded from the study. Each recruited patient underwent a specific questionnaire to evaluate her or his clinical history, including family history of osteoporosis, diabetes and cardiovascular disease and the presence of previous fractures. The level of sedentary lifestyle was assessed through the short version of the International Physical Activity Questionnaires (IPAQ)^15^. Weight, height and BP were also recorded. The body mass index (BMI) was calculated for all subjects. All patients provided informed consent before being enrolled. The study was conducted in accordance with the Declaration of Helsinki. The protocol was approved by the local ethics committee (Comitato Etico Catania 1, Azienda Ospedaliero-Universitaria ‘Policlinico-Vittorio Emanuele’ Catania), with approval number 50 from 15 January 2018.

### Densitometric measurements

As reported in one of our previous studies^16^, BMD was measured in patients and controls by a dual X-ray absorptiometry densitometer (Lunar Prodigy GE Medical Systems), both at the lumbar spine (L1 to L4) in anterior–posterior (A–P) projection and at the femoral neck. The instrument was calibrated on a daily basis according to the manufacturer's instructions. Reproducibility was calculated as a coefficient of variation (CV) obtained by weekly measurements of a standard phantom on the instrument and by repeated measurements obtained in three patients of different ages. The CV of our instrument was 0.5% with the standard phantom; in vivo, we calculated a CV of 1.1% for the lumbar spine and 1.5% for the femoral neck. BMD data are expressed as g/cm^2^.

### Biochemical analyses

In all subjects, blood venous samples were taken in the morning (after 10-h fasting) to measure, by automated routine procedures, calcium corrected for serum albumin (Ca), phosphorus (P), 25-OH vitamin D, parathyroid hormone (PTH), creatinine, total cholesterol, high-density lipoprotein (HDL) cholesterol and triglycerides.

### Haemodynamic study

All participants were studied in a quiet room kept at 22 ± 1 °C after 15 min of recumbent rest, as previously reported^17^. Briefly, a non-invasive haemodynamic study was performed by an experienced operator (LZ) blinded to the clinical data and the therapy. Brachial BP measurements were obtained using an oscillometric device (Dinamap ProCare 100; GE Healthcare, Milwaukee, WI, USA). The cf-PWV (aortic PWV) was measured by a SphygmoCor device (AtCorMedical, Sydney, Australia) using the foot-to-foot velocity method, the intersecting tangent algorithm and the direct distance between the measurement sites^18^: cf-PWV (m/s) = 0.8 × [carotid-femoral direct distance (m)/Δt]. The mean value of two consecutive recordings was used for this analysis. When the difference between the two measurements was ≥ 0.5 m/s, a third recording was performed, and the median value was used. In our laboratory, the intra- and inter-session coefficients of variation of cf-PWV were 3.1% and 6.8%, respectively. The radial pulse wave profile was recorded by applanation tonometry (SphygmoCor system, AtCor Medical) after recalibration with brachial systolic and diastolic BPs in the contralateral arm. It was used to assess the central pulse wave profile and to derive the augmentation index (AIx) and central BP, as previously described and validated^19^.

Longitudinal B-mode (60 Hz, 128 radiofrequency lines) images of the right common carotid artery 2 cm below the carotid bulb were obtained using a high-precision echo tracking device (MyLab Alpha, Esaote, Maastricht, NL) paired with a high-resolution linear array transducer (13 MHz) to acquire cIMT using the built-in echo tracking software. In our laboratory, the intra- and inter-session coefficients of variation of cIMT were 3.3% and 5.9%, respectively.

### Statistical analysis

Continuous variables are presented as the mean ± SD; categorical variables are presented as percentages. The D’Agostino–Pearson normality test was used to confirm that variables were well-modelled by a normal distribution. Clinical and haemodynamic variables were compared using analysis of variance for continuous variables and chi-squared tests for categorical variables. Spearman’s rank correlation coefficient was used to assess the relationship between cf-PWV and femoral neck BMD. A linear regression analysis was performed to study vascular ageing in controls and patients with osteoporosis. A multivariate linear regression analysis was also performed to confirm the association between cf-PWV and BMD. Statistical analyses were performed using NCSS 2007 and PASS 11 software version 07.1.21 (Gerry Hintze, Kaysville, UT, USA; www.ncss.com). A two-tailed p < 0.05 was considered significant.

## Results

**Figure 1:**
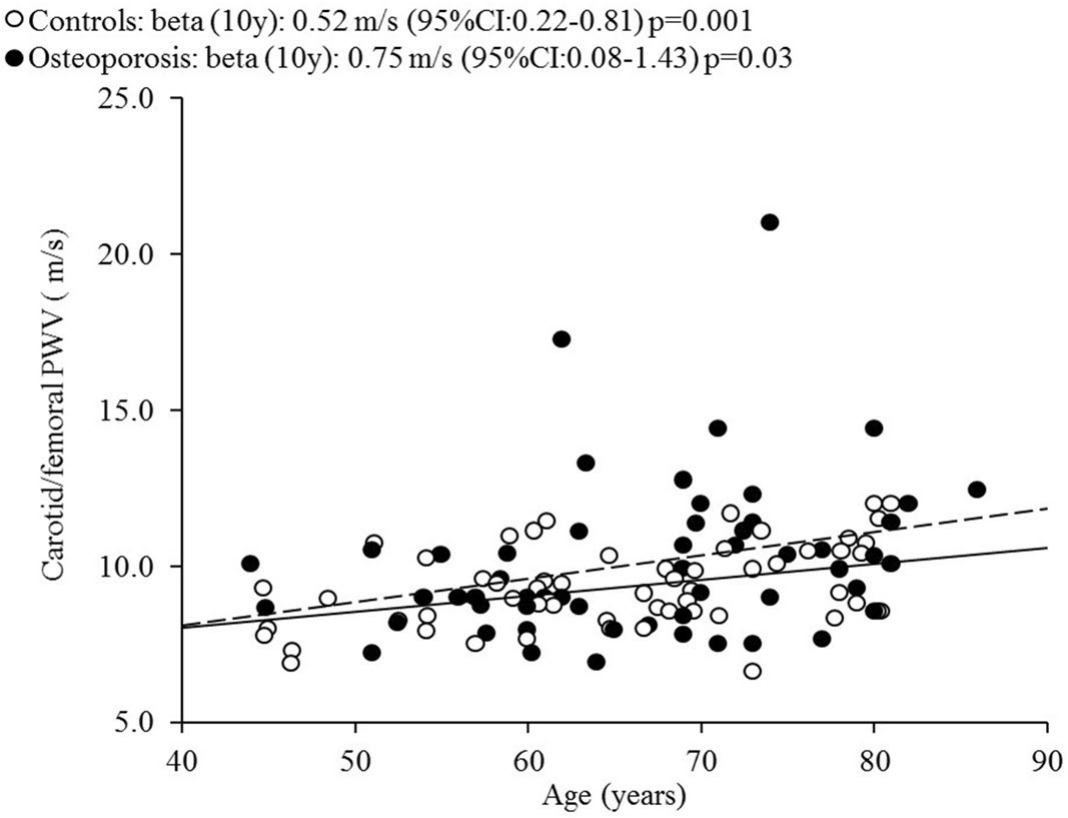
Correlation between carotid-femoral PWV and age. PWV: pulse wave velocity. This figure was created using NCSS 2007 and PASS 11 software version 07.1.21 (Gerry Hintze, Kaysville, UT, USA; www.ncss.com).

**Figure 2:**
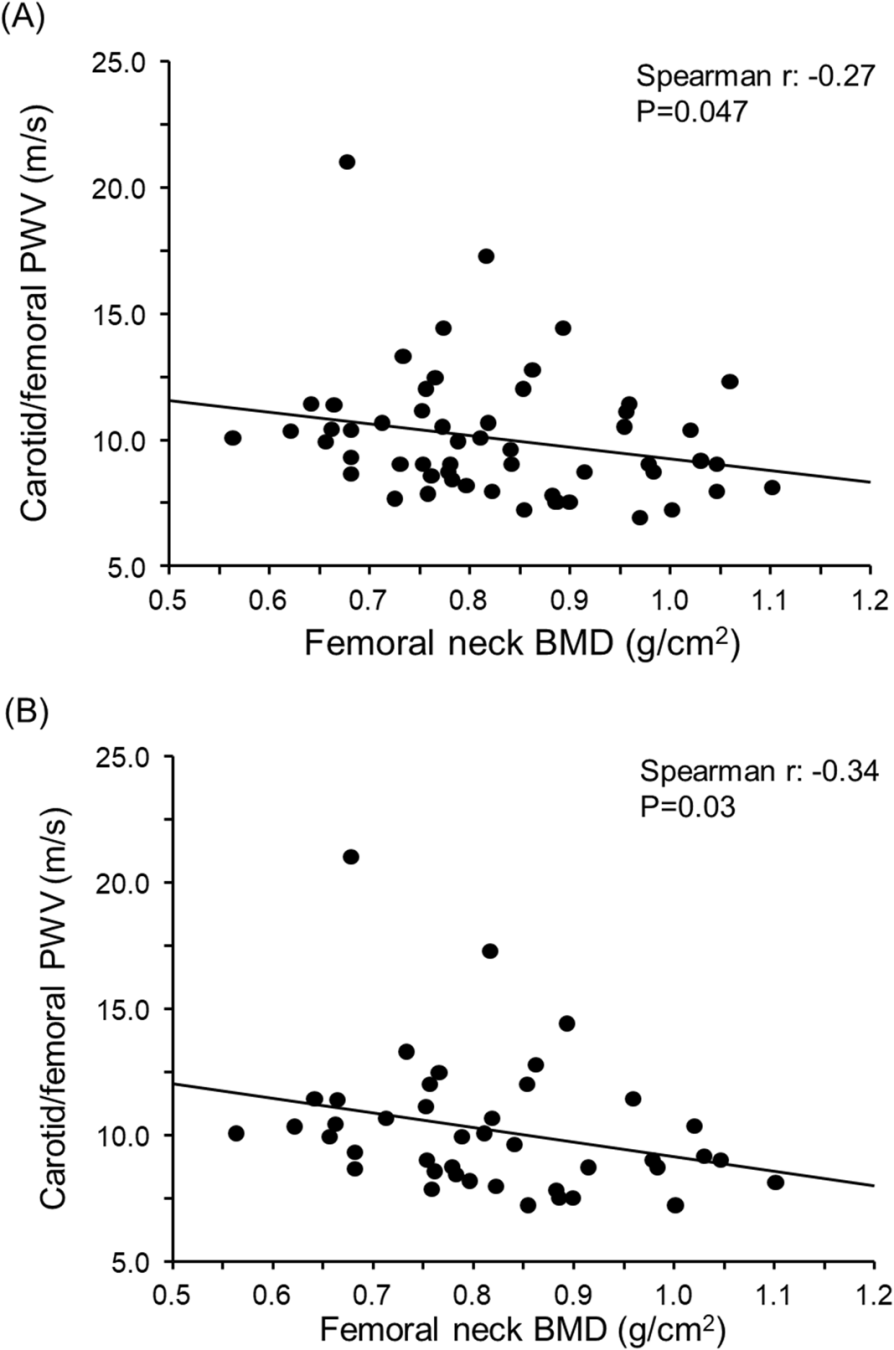
Correlation between carotid-femoral PWV and femoral neck BMD in all patients with osteoporosis (**A**) and in female patients with osteoporosis (**B**). PWV: pulse wave velocity; BMD: bone mineral density. This figure was created using NCSS 2007 and PASS 11 software version 07.1.21 (Gerry Hintze, Kaysville, UT, USA; www.ncss.com).

A total of 58 patients attending the Outpatient Clinic for Metabolic Bone Diseases of the University Policlinic of Catania (Italy) with a T-score ≤ -2.5 SDs were enrolled. An equal number of healthy subjects matched for age was considered the control group. There were no statistically significant differences between the two groups regarding age; BMI; smoking habits; sedentary lifestyle; and total cholesterol, HDL cholesterol and triglyceride levels (Table 1). On the other hand, the percentage of women in the group of patients with osteoporosis who also had slightly higher systolic and diastolic BP values was higher than that in the control group (Table 1). Obviously, patients with osteoporosis had significantly lower lumbar and femoral neck BMD values compared with controls, while there were no significant differences between the two groups regarding the other biochemical parameters. The patients with osteoporosis had a significant increase in cf-PWV, cIMT and AIx compared with controls (Table 2). Cf-PWV values correlated significantly with age in both groups (Fig. 1). Considering only patients with osteoporosis, femoral neck BMD was inversely associated with cf-PWV (beta for 0.1 g/cm^2^ increase: − 0.42 m/s; 95% confidence interval [CI] − 0.79 to − 0.06 m/s; p = 0.047) (Fig. 2A). This result was confirmed even in a multivariate linear regression model adjusted for mean blood pressure and sex (beta for 0.1 g/cm^2^ increase: − 0.44 m/s; 95% CI − 0.88 to − 0.06 m/s; p = 0.02) and in a subgroup of female patients with osteoporosis (Fig. 2B).

**Table 1:** Clinical characteristics and densitometric and laboratory data of patients and controls.

	Controls	Osteoporosis	p-value
n	58	58	–
Age, years	65 ± 11	66 ± 10	ns
Male (%)	58.6	27.5	** < 0.001**
BMI, kg/m^2^	27.0 ± 4.4	27.7 ± 5.4	ns
Smokers (%)	25.8	17.4	ns
Sedentary (%)	18.9	25.8	ns
Previous fractures (%)	0	31	** < 0.001**
Hypertension (%)	31	71	** < 0.001**
Systolic blood pressure, mmHg	128 ± 15	141 ± 15	** < 0.001**
Diastolic blood pressure, mmHg	76 ± 10	81 ± 7	**0.001**
Differential blood pressure, mmHg	52 ± 13	60 ± 15	**0.003**
Mean blood pressure, mmHg	95 ± 10	101 ± 7	** < 0.001**
Lumbar BMD, g/cm^2^	1.123 ± 0.184	1.039 ± 0.241	**0.03**
Femoral BMD, g/cm^2^	0.902 ± 0.134	0.832 ± 0.184	**0.02**
Calcium corrected for albumin, mg/dl	9.3 ± 1.2	9.5 ± 1.3	ns
Phosphorus, mg/dl	3.9 ± 0.8	3.8 ± 0.7	ns
Creatinine, mg/dl	1.0 ± 0.3	0.9 ± 0.3	ns
PTH, pg/ml	46.1 ± 9.6	44.2 ± 8.2	ns
25-OH vitamin D, ng/ml	20.3 ± 5.8	19.3 ± 6.0	ns
Total cholesterol, mg/dl	187 ± 36	180 ± 31	ns
HDL cholesterol, mg/dl	53 ± 15	56 ± 17	ns
Triglycerides, mg/dl	144 ± 30	152 ± 34	ns

Data are expressed, where possible, as the mean ± SD (in parenthesis).

*ns* not significant, *BMI* Body Mass Index, *BMD* bone mineral density, *PTH* parathyroid hormone, *HDL cholesterol* high-density lipoprotein cholesterol.

Significant p-values are in bold.

**Table 2:** Vascular parameters in patients and controls.

	Controls	Osteoporosis	p-value
n	58	58	–
cf-PWV, m/s	9.3 ± 1.3	10.1 ± 2.6	**0.04**
cf-PWV > 10 m/s, %	31	45.5	ns
IMT, µm	710 ± 146	778 ± 150	**0.02**
IMT > 900 µm, %	13.8	18.2	ns
AIx, %	30 ± 13	40 ± 11	** < 0.001**

Data are expressed, where possible, as the mean ± SD (in parenthesis).

*ns* not significant, *cf-PWV* carotid-femoral pulse wave velocity, *IMT* carotid intima-media thickness, *AIx* Augmentation Index.

Significant p-values are in bold.

## Discussion

The present study showed that, in an outpatient population with the same risk factors (apart from a slight increase in BP), patients with osteoporosis had earlier vascular ageing, with an increase in arterial stiffness. In particular, we observed that patients with osteoporosis had higher cIMT, cf-PWV and AIx values than controls. Furthermore, we observed a significant inverse correlation between the femoral neck BMD and cf-PWV values in the whole study population; this finding supports a possible causal role of osteoporosis in vascular ageing. Interestingly, this result was confirmed even after adjustment for major confounders (mean blood pressure and sex). Patients with osteoporosis and controls did not show significant differences for factors that can affect arterial stiffness, such as age, BMI, smoking, physical activity and lipid profile^20,21^, excluding a slight increase in BP. This finding in the group with osteoporosis could be an expression of the increased vascular stiffness observed in these patients.

The association between early cardiovascular risk markers and ‘bone health’ has been the subject of previous studies, with conflicting results. The ‘San Antonio Family Osteoporosis Study’ showed a correlation between a decrease in BMD and an increase in cIMT. In particular, the authors reported a negative association between cIMT and BMD in both men and women over 60 years old^22^. Other studies have confirmed this correlation, but only in postmenopausal women^23,24^. Shin et al.^25^, in a Korean population, did not observe a correlation between cIMT and BMD in men and postmenopausal women. Kim et al.^26^ reported an inverse relationship between cIMT and BMD, but only in women with acute stroke.

The data on arterial stiffness are also partially conflicting, particularly in men. In a previous study at our centre, AIx and cf-PWV were significantly higher in postmenopausal women compared with controls^27^. In a study involving around 2500 Chinese patients, Wang et al.^28^ showed a strong correlation between brachial-ankle PWV and lumbar BMD in both sexes. By contrast, a study of 332 Dutch men observed no correlation between cf-PWV, AIx and BMD^29^.

Only a few longitudinal studies have assessed the relationship between BMD and arteriosclerosis. Jaalkhorol et al.^30^ recently observed, in a female Japanese population, that low BMD for the total hip was significantly associated with the development of increased arterial stiffness during a 10-year follow-up.

Arterial stiffness is largely determined by structural changes in the vessel walls, such as those associated with age-related atherosclerosis and/or arteriosclerosis^31^, but the mechanical properties of the great arteries are also regulated by the endothelium through the production and release of nitric oxide (NO). Endothelial function has been reported to be independently and inversely associated with cf-PWV and AIx in healthy individuals^32^. It has been recently shown that postmenopausal women with osteoporosis without important risk factors for cardiovascular disease had impaired brachial artery endothelial function compared to postmenopausal women with normal BMD or osteopenia^33^. Therefore, impaired endothelial function may be responsible for alterations in sphygmic wave reflection and arterial stiffness in postmenopausal osteoporosis. Menopause has been reported to increase arterial stiffness^34^, a phenomenon that suggests oestrogen deficiency plays a causal role. In addition, hormone replacement therapy has been shown to reduce PWV in postmenopausal women, possibly through a vasodilator mechanism and/or by modulating the relative proportions of collagen and elastin and the number of smooth muscle cells in the vessel wall^35^.

In recent years, great attention has been focused on the role of WNT/ß-catenin signalling^12,36^ and the OPG/RANK/RANKL system^13,27,37^ with regard to the association between osteoporosis and vascular stiffness. They seem to be involved, but it is not currently clear whether they have a pathogenetic role or only represent markers of subclinical arteriosclerosis.

Our study also has some limitations: the small number of patients and transverse nature of the study do not allow the determination of the relationship between the progression of carotid atherosclerotic disease and bone remodelling. The difference in gender distribution between patients with osteoporosis and controls present in our study should not have affected our results because a negligible influence of gender on PWV (0.1 m/s difference) had been previously observed^38^. Moreover, the association between low femoral neck BMD and high cf-PWV was confirmed even after adjustment for sex and in a subgroup of females with osteoporosis.

In conclusion, we showed that an outpatient population consisting of men and women with osteoporosis exhibited an increase in arterial stiffness and cIMT, two early markers of cardiovascular risk. Prospective studies are needed to improve the understanding of the pathophysiological mechanisms that correlate arterial stiffness with the bone remodelling process. Comprehending the pathophysiological links that correlate osteoporosis with atherosclerotic processes could allow, in the near future, the identification of drugs that could concomitantly treat osteoporosis and have a beneficial effect on vasculature and bone. This could determine an improvement in the patients’ quality of life and the discovery of cost-effective preventive and treatment regimens for these two pathological conditions.
